# Clean-Up of Heavy Metals from Contaminated Soil by Phytoremediation: A Multidisciplinary and Eco-Friendly Approach

**DOI:** 10.3390/toxics11050422

**Published:** 2023-05-02

**Authors:** A. K. Priya, Muthiah Muruganandam, Sameh S. Ali, Michael Kornaros

**Affiliations:** 1 Department of Chemical Engineering, KPR Institute of Engineering and Technology, Coimbatore 641407, India; 2 Project Prioritization, Monitoring & Evaluation and Knowledge Management Unit, ICAR-Indian Institute of Soil & Water Conservation (ICAR-IISWC), Dehradun 248195, India; mail2mmm20@gmail.com; 3 Biofuels Institute, School of the Environment and Safety Engineering, Jiangsu University, Zhenjiang 212013, China; samh@ujs.edu.cn; 4 Botany Department, Faculty of Science, Tanta University, Tanta 31527, Egypt; 5 Department of Chemical Engineering, University of Patras, 1 Karatheodori Str., University Campus-Rio, 26504 Patras, Greece

**Keywords:** biotechnological methods, genetic modifications, heavy metal degradation, phytoremediation, remediation techniques

## Abstract

Pollution from heavy metals is one of the significant environmental concerns facing the world today. Human activities, such as mining, farming, and manufacturing plant operations, can allow them access to the environment. Heavy metals polluting soil can harm crops, change the food chain, and endanger human health. Thus, the overarching goal for humans and the environment should be the avoidance of soil contamination by heavy metals. Heavy metals persistently present in the soil can be absorbed by plant tissues, enter the biosphere, and accumulate in the trophic levels of the food chain. The removal of heavy metals from contaminated soil can be accomplished using various physical, synthetic, and natural remediation techniques (both *in situ* and *ex situ*). The most controllable (affordable and eco-friendly) method among these is phytoremediation. The removal of heavy metal defilements can be accomplished using phytoremediation techniques, including phytoextraction, phytovolatilization, phytostabilization, and phytofiltration. The bioavailability of heavy metals in soil and the biomass of plants are the two main factors affecting how effectively phytoremediation works. The focus in phytoremediation and phytomining is on new metal hyperaccumulators with high efficiency. Subsequently, this study comprehensively examines different frameworks and biotechnological techniques available for eliminating heavy metals according to environmental guidelines, underscoring the difficulties and limitations of phytoremediation and its potential application in the clean-up of other harmful pollutants. Additionally, we share in-depth experience of safe removing the plants used in phytoremediation—a factor frequently overlooked when choosing plants to remove heavy metals in contaminated conditions.

## 1. Introduction

Increasing industrialization and agricultural practices have led to the widespread contamination of soil with heavy metals (HMs), which has become a significant environmental concern globally [1,2]. HMs are exceptionally stable entities with half-lives that are more than 20 years long [3,4]. The extraction of minerals and associated various handling techniques influence the introduction of pollutants and HMs into the environment, which largely determines the specific mobility of different metals in the environment. Due to the expansion in industrialization and the organic cycle’s unsettling influence, heavy metal contamination becomes a problematic issue that needs an effective solution to moderate its effects. Heavy metals are essentially large, non-biodegradable metals that accumulate in the environment and pose a risk to human and environmental health by contaminating soil and water, for example [5]. In a living organic entity, these elements accumulate in the body tissue through a process known as bioaccumulation, and they move from a lower to a higher trophic level with an elevated concentration—a phenomenon known as biomagnification. Due to the negative effects of heavy metals, there are fewer soil organisms in the soil [6]. 

Due to the fundamental role of soils in biological solidity and horticultural creation, they are considered as essential components of earthly environments [7,8]. However, excessive mining operations, rapidly expanding modern activities, waste removal, and careless use of engineered composts and pesticides have made soil tainting by potentially toxic elements (PTEs) a significant natural issue globally [9,10,11]. Heavy metals are often defined as metals or metalloids with a higher atomic number and atomic weight than lighter elements, such as carbon, nitrogen, and oxygen. They are typically characterized by a density greater than 5 g/cm³ and a thickness that exceeds a certain threshold [12,13]. Ali et al. (2019) proposed an alternative definition of heavy metals based on nuclear density, specifically a nuclear density of more than 41 g/cm^3^ [14]. They used specific metals, including Cd, Cr, Co, Cu, Pb, Hg, and Zn, as examples of heavy metals based on their nuclear density. Heavy metals can also accumulate in the food chain, potentially leading to health problems in animals and humans who consume contaminated food. Heavy metal contamination is a widespread issue affecting millions of hectares of land worldwide. Expanded persistence through varying rates, land degradation, and geological cycles are the leading recurring causes of soil contamination or depletion [15]. The effects of excessive exposure to soil toxins, such as heavy metals, on plant development and physiological cycles include reducing seed germination [16], limiting plant growth [17], disrupting nutrient uptake [18], stifling photosynthesis [19], and adjusting enzymatic activities [20]. PTEs cause higher plants to become more susceptible to oxidative damage by producing excess reactive oxygen species (ROS) [21]. In addition, soil PTEs can increase risks to human health as they rise in the food chain [22,23,24].

Studies have shown that the primary source of lead and cadmium in the local air is the release of gases from gas fuel, brakes, and tires [25]. Subsequently, HMs, such as Copper (Cu), Lead (Pb), Zinc (Zn), Nickel (Ni), Cadmium (Cd), Chromium (Cr), Iron (Fe), Manganese (Mn), and natural toxins, such as hydrocarbons, sweet-smelling compounds, phenols, organo-chlorinated compounds, and pesticides, have been found at higher levels in contaminated soil [26]. The first category, which focuses on improving the ability of plants to remediate heavy-metal-polluted soils, highlights the importance of identifying plant species and genetic traits that effectively remove heavy metals from the soil. This research is essential for developing efficient and sustainable phytoremediation strategies. The second category, which analyses the effects of heavy metals on plants from a molecular perspective, is necessary for understanding the mechanisms underlying plant responses to heavy metal stress. This knowledge is essential for identifying molecular targets that can be manipulated to enhance plant tolerance and phytoremediation efficiency. The third category, which focuses on the progress of the plant remediation effect on heavy metal pollution, is critical for evaluating the effectiveness of phytoremediation in the field. These studies provide important insights into the factors that influence the success of phytoremediation, such as soil properties, plant species, and environmental conditions [27].

Food crops contaminated with HMs have the potential to bioaccumulate HMs, and the ensuing biomagnification along the higher trophic levels in the established pecking order can potentially harm the prosperity of both living things and people [5]. HM development in the body can bring about unexpected severe problems, for example, *itai* sickness due to diligent disc openness; kidney harm and ailment because of Zn; mucosa disintegration, hepatic structure disappointment, and central tactile framework harm because of Cu; and skin aggravation and tangible framework entrapments because of Ni [28].

These heavy metals have toxicological effects that can reduce the number of living things and their productivity in the soil where they are found on Earth’s surface. The use of vegetation for natural remediation is an ancient practice that cannot be pinned down to a single institution; however, a line of fascinating logical developments combined with an interdisciplinary review methodology has allowed the extension of this knowledge into powerful biological hardware, known as phytoremediation. In which, green vegetation is suitably used to remove impurities, rendering the contaminants in the environment innocuous or consolidated for their easy elimination [29]. 

Soil recovery from heavy metal pollution is possible, but the methods used depend on the specific metals present, their speciation, and the degree of contamination. Possible approaches include physical and chemical treatments, such as soil washing, electrokinetic remediation, and phytoremediation. The effectiveness of these methods will depend on factors such as the type of soil, the nature of the contaminants, and the desired end-use of the remediated soil. Risk assessment, which contributes to the modest protection of the environment and general wellbeing, is an effective tool that enables strategists and decision makers to manage heavy-metal-polluted regions [11]. If soils polluted with pesticides are used to grow food crops, there is a risk that these pollutants will enter the food chain and potentially harm humans and animals that consume the contaminated crops. Pesticides can persist in the soil for long periods, especially if natural processes do not break them down or if they are continuously applied. The pesticides can then be taken up by the roots of food crops and accumulate in the edible parts of plants, such as fruits, vegetables, and grains [30].

**Figure 1:**
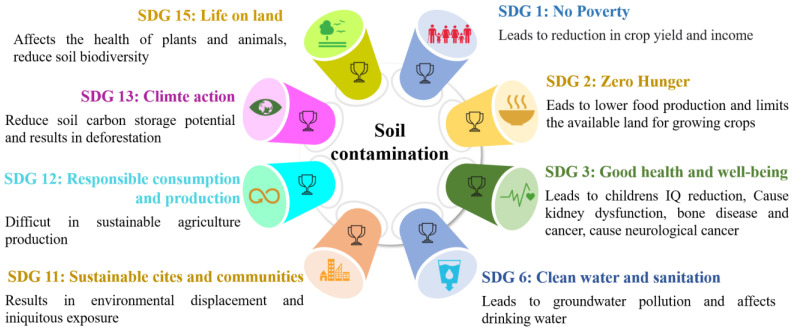
Main effects of soil contamination on sustainable development goals [33].

Phytoremediation, impurity immobilization, and soil washing are commonly known techniques that are effective for the remediation of metal-contaminated soil [31]. Despite their efficiency and eco-friendly nature, these innovations have not yet been widely used in non-industrial nations due to inadequate knowledge or awareness. However, major realistic applications of these advancements have been considered effective and applied in developed nations. Additionally, effective communication and community engagement are crucial for ensuring the success of soil remediation efforts. It is essential to involve local communities in designing and implementing soil remediation strategies and to provide them with information about the risks associated with contaminated soil and the potential benefits of remediation [32]. The main effects of soil contamination on sustainable development goals are shown in Figure 1.

This study examines both rural and urban soils and includes an overview of the potential of plant species with capacity for accumulating contaminants, known as hyperaccumulator plants, tools and approaches required for phytoremediation, and biotechnological strategies for removing heavy metals. Additionally, the study explores in situ and ex situ remediation approaches for heavy-metal-contaminated soil, focusing on phytoremediation methods. A critical aspect of the study is the examination of the effects of heavy metals on food security, including their potential for bioaccumulation and biotoxicity. The study also explores potential ways of removing heavy metals from soil using phytoremediation techniques and possible applications of the byproducts of phytoremediation. This study provides a comprehensive overview of recent developments in removing heavy metals from contaminated soil using phytoremediation techniques and the potential consequences for food security and other human and ecological health aspects. The study also explores possible uses of phytoremediation products and proposes ideas for future innovations in this field. This study provides a comprehensive and forward-thinking analysis of phytoremediation and its potential implications.

## 2. Heavy Metal Sources in the Environment

**Figure 2:**
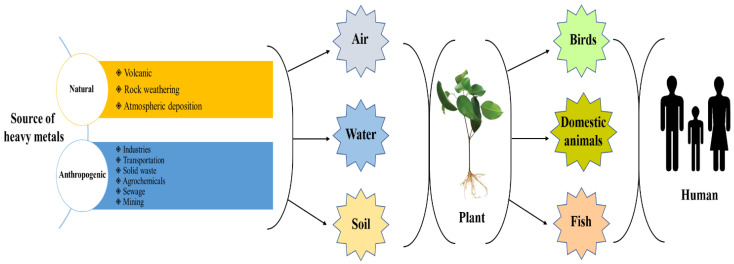
Various sources of heavy metals and their environmental pathways.

Due to the potential for bioaccumulation and poisoning of living organic entities, HM contamination has drawn attention across the world [28]. The effects of HM contamination in the environment significantly impact biological systems both in terrestrial and marine ecosystems [34]. In addition to diffuse sources, point sources of contamination, such as mining, smelting, and manufacturing, can also contribute to heavy metal contamination in soil and water. These activities can release high concentrations of heavy metals into the environment and can result in localized contamination of soil, water, and air [31]. These water contaminations are most dangerous and harmful to human and natural environments [28]. Various sources of heavy metals and their environmental pathways are shown in Figure 2. 

Heavy metal contamination of soil can arise from both natural and anthropogenic sources. Heavy metals in contaminated areas can typically be obtained from parent soil, also referred to as a lithogenic source. Numerous heavy metals do not exist separately; instead, they exist as synthetic structures that can be readily and directly absorbed by living cells and tissues [35]. Zn, Hg, Cd, Pb, Cu, Ni, As, and Cr are among the most commonly found heavy metals in soil. Other heavy metals, such as aluminum (Al), barium (Ba), cobalt (Co), manganese (Mn), selenium (Se), and silver (Ag), can also be present in soil at elevated levels and can pose environmental and health risks. Natural events, such as volcanic emissions, ocean salt sprays, wind-borne soil particles, forest fires, and rock weathering, can contribute to the presence of heavy metals in soil. Biogenic sources, such as the decay of organic matter, can also release heavy metals into soil [36].

Anthropogenic activities, such as industrial processes, mining, and agriculture, can significantly increase the presence of heavy metals in soil beyond natural levels, leading to potential environmental and health hazards. Hazardous materials, such as As, Cd, Cr, Cu, Hg, Pb, and others, can be found in various sources, including sewage, paints, alloys, electronic products, and wastewater from mines. These materials can easily leach into soil and accumulate over time, contaminating soil [37]. Heavy metal contamination can sometimes result from accidental spills or leaks from industrial sites or transportation. The origins of heavy metal pollution in soils vary and can be attributed to natural and human activities [38]. For instance, the E-waste incinerator site in East Jerusalem was studied on soil samples taken from 29 different locations specified varying mobility of heavy metals [39]. These activities can release heavy metals into the air, water, and soil, leading to contamination and potential health hazards for humans and wildlife. Therefore, effective management and control of anthropogenic sources are necessary to reduce environmental heavy metal pollution.

## 3. Recent Developments in Heavy Metal Remediation Strategies

**Figure 3:**
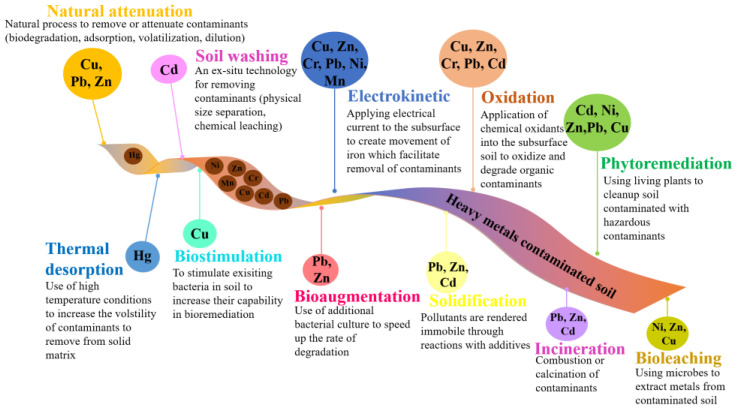
Remediation techniques for heavy metal removal.

Soil contamination by heavy metals has become a growing concern due to rapid urbanization and industrialization. Heavy metals, such as lead, arsenic, cadmium, and mercury, are persistent and toxic in the environment, and they can accumulate in the soil over time. This contamination can seriously affect the environment and human health [40]. Soil remediation (Figure 3) is thus a critical process to help protect the environment and human health. Soil can become contaminated with harmful substances, such as heavy metals, pesticides, and petroleum products, through industrial and agricultural practices, waste disposal, and accidental spills [41].

A soil remediation technique known as soil substitution reduces the concentration of pollutants in to a permissible natural limit. Soil spading and soil substitution are two methods used in soil remediation to mitigate the effects of soil contamination by heavy metals or other pollutants. Soil spading, also known as soil turning or soil tilling, involves digging and mixing the polluted soil with pure soil to reduce contaminant concentrations. This method is often used for large areas of contaminated soil, where removing and replacing the entire contaminated soil layer is impractical. Soil spading helps to aerate the soil and promotes microbial activity, which can accelerate the natural breakdown of contaminants in the ground. Soil substitution, also known as excavation and disposal, is a method of soil remediation that involves removing contaminated soil and replacing it with pure soil. This approach is often used for small, localized areas of contamination, such as around underground storage tanks or industrial sites. Soil substitution can effectively reduce the risk of exposure to the contaminant and restore the soil to a healthy state. Both ground spading and soil substitution can be effective methods for soil remediation, but they each have advantages and disadvantages.

The choice of method will depend on the nature and extent of the contamination and other factors, such as cost and accessibility. It is essential to carefully assess the situation and consult with experts to determine the best approach for remediation [35]. This method calls for treating the replaced soil to stop further contamination [42]. Another process of making the contaminated soil less potent is adding new ground and covering the old. This technique effectively reduces the climate-damaging effects of toxic emissions, but it is costly, necessitates a sizable working area, and is only suitable for treating soil that is locally severely contaminated. Furthermore, carrying out earthworks while utilizing the strategy for replacing the soil may also disturb the local environment [43].

Thermal desorption is used in soil remediation to volatilize heavy metals and metalloids, such as mercury and arsenic, from contaminated soil. While thermal desorption is a method of soil remediation that involves heating contaminated soil to release the contaminants, it does not necessarily involve the use of microwaves, steam, or infrared radiation. Once the heavy metals have been volatilized, a negative vacuum tension is applied, or a transporter gas is used to collect and remove the volatile heavy metals from the contaminated soil. Two types of thermal desorption methods exist: high-temperature desorption and low-temperature desorption. High-temperature desorption is typically performed at temperatures between 320 and 560 °C and is used for heavily contaminated soils. On the other hand, low-temperature desorption is performed at temperatures between 90 and 320 °C and is used for less contaminated soils [35,42]. This innovation has the advantage of being a simple technique for soil remediation. However, the equipment needed to complete thermal desorption is expensive and the process takes a long time [43]. 

Soil washing typically involves mechanical agitation of the soil using water or aqueous solutions containing chelating agents and/or surfactants. The chelating agents and surfactants help to solubilize and mobilize contaminants, such as heavy metals, pesticides, and petroleum hydrocarbons. The washing process generates a wastewater stream or leachate that contains the dissolved contaminants. The wastewater is then treated using a range of technologies, such as chemical precipitation or biological treatment, to remove or immobilize the contaminants before discharge into the environment [11]. Soil washing typically involves several stages of physical and chemical processes to remove contaminants from soil. The soil is first physically washed to remove large debris and impurities; then, it undergoes several chemical extraction stages to remove the remaining contaminants. The extracted contaminants are treated and disposed of separately, and the cleaned soil can be returned to its original location [44]. 

Electrokinetic remediation is another method used for soil remediation to clean up contaminated soil. It involves using an electric field gradient to remove heavy metals and other contaminants from the soil. This method involves placing electrodes in the soil and passing a direct current through the soil between the electrodes. This creates an electric field gradient that drives the movement of heavy metals and other contaminants from the soil toward the electrodes [45]. The contaminants are then removed from the soil by a process known as electroosmosis, where a liquid solution is drawn through the soil by the electric field, carrying the contaminants with it. Electrokinetic remediation has some advantages over other soil remediation methods. It is particularly effective for heavy metals and other ionizable contaminants and can be used for various soil types [44]. Additionally, it does not require the removal of contaminated soil, which can be beneficial for preserving the site’s integrity [35,42]. In this method, two cathodes are embedded in the degraded soil to produce a weak electric field [35]. The primary way of transporting foreign materials from soil to anodes is done through electromigration, electrophoresis, or electroosmotic streaming. When the contaminant adsorbs or advances at the anode, it can be removed. This method is practical for treating low-vulnerability soil, requires little effort, and is simple to use [46]. Since it does not disrupt the typical biological system in the soil, this treatment method is considered safe for the ecosystem. However, the treatment effectiveness of this approach is low, and it has no effect on the pH value of the soil. A longer-term investment in remediation is required if interlaced or covered metal objects are present in the soil, because they will cause the current stream to divert. The high concentration of obscure toxins will also affect the viability of electrokinetic remediation. 

**Figure 4:**
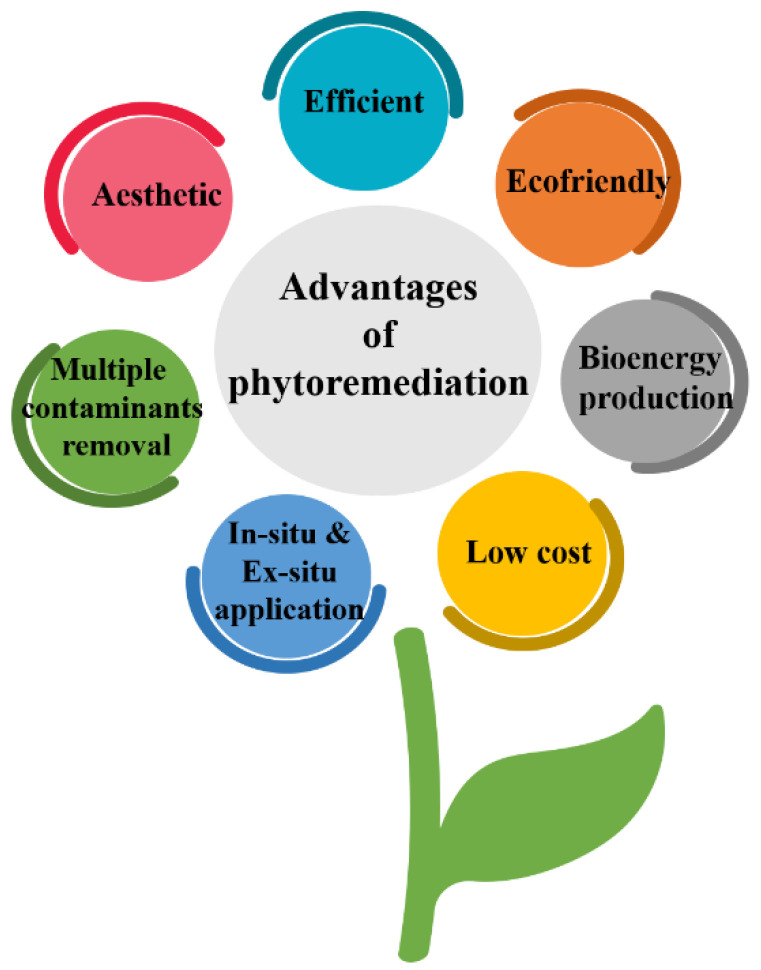
Advantages of phytoremediation techniques.

Phytoremediation is a promising soil remediation technique that has gained attention from various experts due to its efficiency, cost-effectiveness, and eco-friendly nature. It involves using plants to remove or degrade contaminants from the soil [44]. The advantages of phytoremediation techniques are shown in Figure 4.

## 4. Heavy Metal Removal from Contaminated Soil by Phytoremediation

Phytoremediation uses metal-aggregating plants to reestablish debased natural resources, namely, soil and water [47]. The uptake of heavy metals from contaminated soil can occur over the course of several distinct cycles during phytoremediation. The specific system depends on the type of toxin, the plant species used, and other ecological factors. In a process known as phytostabilization, plants are used to immobilize pollutants in soil and prevent their spread to new areas. Plant roots release proteins and organic acids into the soil during the rhizodegradation cycle, which separates soil impurities. Phytoextraction involves the uptake of contaminants by plant roots and their accumulation in the above-ground plant tissues [36]. Phytodegradation is the process by which plants break down contaminants in their tissues through metabolic processes [44]. Phytoaccumulation is when plants take up contaminants and store them in their tissues without breaking them down. Phytovolatilization is when plants release contaminants into the air through transpiration or other mechanisms [36]. Each of these processes has advantages and limitations, and the best approach depends on the specific situation. For example, phytoextraction may be effective for removing high concentrations of contaminants from the soil, while phytostabilization may be more appropriate for lower concentrations of contaminants or for preventing their spread to other areas [48].

The removal of heavy metals from soil and water and the selection of suitable plant species can both be accomplished through phytoremediation [49]. Plants are viable options for phytoremediation due to their physiological capacity to withstand and accumulate heavy metals as well as their adaptability to various environmental conditions. Furthermore, the use of specific plant species for phytoremediation can be tailored to the type of heavy metal pollution, as different plant species have varying abilities to accumulate specific heavy metals [50]. There are various techniques that have been developed for the removal of heavy metals through phytoremediation processes.

However, compared to physiological strategies, they are less viable and more expensive [51]. Standard techniques, such as physicochemical cycles, are typically used to remove contaminants from soil. Due to their more notable effectiveness and lower costs, physiological strategies are generally recognized as additional promising options for soil remediation [31]. Bioremediation can also involve using microorganisms to break down or transform contaminants into less toxic forms. Microorganisms can degrade organic pollutants or transform heavy metals into less harmful substances. For example, some bacteria can convert poisonous forms of mercury into less toxic elemental mercury, which can then be released into the atmosphere. Other microorganisms can break down organic pollutants, such as petroleum hydrocarbons, into harmless byproducts. Phytoremediation is a plant-based method that uses plants to absorb and remove contaminants from soil or water [52]. Plants with high phytoremediation capabilities are often characterized by fast growth, high biomass, deep roots, and efficient uptake and transport of heavy metals or other contaminants to their above-ground parts. The use of phytoremediation can be a cost-effective and environmentally friendly approach to remediate contaminated sites [53].

**Figure 5:**
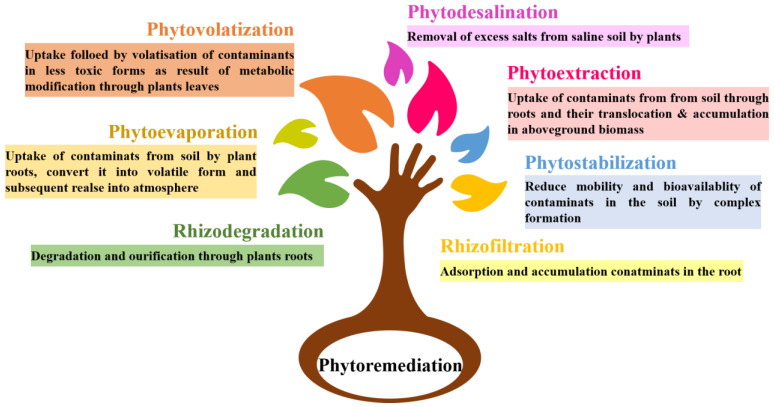
Techniques for phytoremediation.

Due to its unique ability to eliminate dangerous synthetic substances through plant underground root growth, bioaccumulation, impurity debasement, or movement [54], the phytoremediation approach has many advantages in ecological clean-up. Various techniques for phytoremediation are shown in Figure 5. Using phytoremediation for heavy metal removal from polluted soils can also allow for the cultivation of oil crops for biodiesel or bioenergy production. In a study on the use of sunflower and canola crops for the phytoremediation of heavy-metal-contaminated soils, it was found that not only did the plants reduce heavy metal concentrations in the soil, but they also produced high yields of oil for use in biofuel production [55]. This suggests that phytoremediation could be valuable for environmental remediation and sustainable energy production [56]. Table 1 shows the potential plant species which can be used for heavy metal remediation. 

### 4.1. Phytoextraction

This process involves using the natural ability of plants to absorb and accumulate pollutants through their roots and above-ground parts and transform or degrade them into less harmful substances. The plants uptake these pollutants through their root systems and then transport them to their above-ground biomass, accumulating them in various plant parts, such as leaves, stems, and fruits. In some cases, the pollutants can be removed from a site by harvesting the plants and disposing of them elsewhere. The plants can be used for various purposes, such as biofuel production or livestock feed, provided the accumulated toxins are safe. This process is referred to as phytoextraction [75]. Metal exchange to shoots is a significant physiological interaction because projections are much easier to collect than roots—the most effective phytoremediation method for removing heavy metals and metalloids. The main idea behind phytoextraction is to grow appropriate plant species on site, collect the metal-enriched biomass, and then treat it to reduce its mass and size [49].

The collected biomass can be processed in several ways, such as fertilizing the soil, drying it out, and heating it until it disintegrates. This process is called “phytomining”, and it is used to extract metals, such as nickel, cobalt, and zinc, from the enriched plant material. However, phytoextraction is not always economically feasible, especially for metals such as lead and cadmium, which have a lower market value [76]. *Lemna valdiviana* is a species of duckweed that is native to North America and is known for its ability to accumulate heavy metals and metalloids, such as arsenic, in its tissues. *Lemna valdiviana* can actually remove up to 82% of arsenic from contaminated water, according to studies on the plant [77]. This makes it a promising candidate for the phytoremediation of arsenic-contaminated water sources. However, further research is needed to fully understand the plant’s arsenic accumulation mechanisms and optimize its use in phytoremediation applications [77]. *Bixa orellana* and *Zea mays* L. are exciting examples of plants with high phytoremediation potential for heavy metals, particularly chromium and lead. It is also noteworthy that adding chelating agents, such as ethylenediaminetetraacetic acid (EDTA), can enhance the phytoextraction process by increasing the solubility and availability of heavy metals in soil [78]. A study on lettuce species and their ability to accumulate copper is also valuable in identifying plant species that can effectively remove specific heavy metals from contaminated soils [79].

### 4.2. Phytostabilization 

Phytostabilization is a phytoremediation technique that uses metal-tolerant plant species to immobilize heavy metals in soil and reduce their bioavailability. This is achieved using plants with deep root systems that can penetrate and stabilize the soil, preventing heavy metals from leaching into the environment [80]. This can occur through various mechanisms, including precipitation or complexation of heavy metals in the rhizosphere, uptake, and accumulation of heavy metals in root tissues, and adsorption onto root cell walls. The immobilized heavy metals become less mobile and bioavailable, reducing the potential for human and environmental exposure [81]. Phytostabilization is a technique that helps preserve soil health at heavy-metal-contaminated sites. It involves using plants to immobilize heavy metals in the soil and prevent their dispersion by wind or runoff. This technique is advantageous because it does not require the removal of contaminated biomass, unlike phytoextraction [43]. To achieve effective phytostabilization, appropriate plant species tolerant to heavy metal conditions must be selected, have dense root systems, and be able to produce a significant amount of biomass. Additionally, soil amendments adjust metal speciation, reduce metal solubility and bioavailability, and improve the physicochemical and biological properties of soil. These amendments can include organic or inorganic materials that increase soil organic matter content and essential nutrients, improving plant colonization and water-holding capacity [82].

### 4.3. Phytovolatilization 

This technique eliminates poisons from soil without requiring the land to be taken away or dealt with. Nonetheless, the viability of phytovolatilization relies upon the toxins present, the plant species utilized, and natural conditions. For example, in the phytovolatilization of Hg, the vaporized Hg can be recondensed and redeposited in the environment, resulting in water and soil pollution. Therefore, phytovolatilization must be utilized cautiously, and ecological conditions, for example, wind speed and direction, must be assessed to limit potential ecological harm [55]. Phytovolatilization is viable in controlling certain environmental pollutants, yet it is not broadly utilized as it has certain limitations. The fundamental disadvantage is the likelihood of airborne toxins causing pollution in surrounding areas. Hence, it is necessary to use this strategy cautiously and only in areas with low population densities or air contamination restrictions exist [44].

### 4.4. Rhizofiltration

Rhizofiltration is a promising approach for removing contaminants from water and liquid waste, and several plant species have been identified as effective in this process. Plants with fibrous root systems and large surface areas are well-suited for rhizofiltration [28]. For example, *Typha latifolia* effectively removes methyl parathion from hydromorphic soils, while bean species (*Phaseolus vulgaris*) have been found to extract uranium and cesium from groundwater efficiently. *Arundo donax* is effective in rhizofiltrating copper from constructed wetlands, and *Eichhornia crassipes*, *Salvinia molesta*, and *Pistia stratiotes* have been identified as promising options for heavy metal removal from industrial sludge [83]. Rhizofiltration has the advantage of being relatively low-cost and environmentally friendly compared to other remediation methods. However, significantly reducing contaminant levels may take longer than other methods, such as chemical treatments or excavation [36].

### 4.5. Rhizodegradation

Rhizodegradation is a natural and cost-effective method for the remediation of contaminated soils. It is a complex process involving interaction between plant roots, microorganisms, and contaminants [51]. Rhizodegradation is a type of phytoremediation that consists in using plants and their associated root-zone microorganisms to degrade pollutants in soil. The rhizosphere is the zone of soil surrounding the roots of plants, where there is a high concentration of microorganisms that can interact with the plant and the contaminants. The selection of plant species also plays a crucial role in rhizodegradation, as different plants release different types and quantities of exudates, which can influence the microbial community and their ability to degrade contaminants. Rhizodegradation has several advantages over traditional remediation methods, including its low cost, reduced environmental impact, and potential for long-term effectiveness [41]. However, the efficiency of the process depends on several factors, such as the type of contaminants present in the soil, the type of plants used, and the environmental conditions. The effectiveness of rhizodegradation can depend on selecting appropriate plants and microorganisms for a particular site. Different plant species and their associated root-zone microorganisms can degrade different types of pollutants and survive and thrive in different environmental conditions [84].

### 4.6. Phytodesalination 

Phytoremediation has been widely studied and accepted to remove salt from impacted soils using halophytic plants [85]. Halophytes are plants that are adapted to grow in highly saline environments, and they are more effective in heavy metal conditions than glycophytic plants, which grow in non-saline environments [86]. Studies have shown that halophytic plants, such as *Suaeda maritima* and *Sesuvium portulacastrum*, can remove 504 kg and 474 kg of salt from a 1-hectare salt-impacted field in just four months [87]. These plants can accumulate sodium chloride from highly saline soils, which helps improve crop yield and quality. Additionally, this method helps reduce soil salinity levels, allowing glycophytic crops, such as *Hordeum vulgare*, to grow normally. Overall, phytoremediation using halophytic plants is a promising strategy for remediating salt-impacted soils, improving crop yield, and reducing the negative impacts of salinity on plant growth [6].

## 5. Potential Biotechnological Approaches for Phytoremediation

Several aids or techniques can enhance phytoremediation, depending on the specific contaminants and environmental conditions. Some of these aids include: Bioaugmentation: This involves adding beneficial microorganisms to the soil, which can help to break down contaminants and improve plant growth [56]. Phytoextraction: Utilizing plants that can accumulate large concentrations of pollutants in their tissues allows for secure collection and disposal. Rhizofiltration: This technique uses the roots of plants to filter contaminants from water. The roots absorb the contaminants and then release them into the plant tissue, where they can be broken down or stored [31]. Hyperaccumulation: This method makes use of plants that can store exceptionally large concentrations of specific pollutants in their tissues. These plants typically remove metals, such as nickel or lead. Soil amendments: Adding certain substances to soil, such as activated carbon or organic matter, can help to improve the soil structure and increase the availability of nutrients for the plants. Genetic engineering: Researchers can modify plant characteristics to increase their ability to remove specific pollutants, as heavy metals, in our case [45].

Plant biotechnology techniques undoubtedly contributed to the development of transgenic crops, and researchers have been working toward the development of efficient, ethical, and cost-effective bioremediation techniques; however, there are still some challenges. Since they differ from physical–synthetic remediation techniques, these biotechnologies have emerged as alternative options aiming for natural rehabilitation. Other intrusive techniques include high-temperature vitrification, corrosive washing, and the removal of soil from an area, all of which have higher associated costs and have an impact on soil productivity and biodiversity [88]. From a molecular viewpoint, a successful candidate for phytoremediation should have an adequate root-uptake genetic system that allows it to take up pollutants from soil efficiently. The plant should also have a high degree of root-to-shoot movement so that the contaminants can be transferred from the roots to the above-ground portions of the plant. The ability of a plant to withstand xenobiotic stress is influenced by several traits, including those that encrypt detoxification catalysts and carriers that transport the toxin out of the cell. Some important genes include phytochelatin synthase, which synthesizes phytochelatins, which bind to heavy metals and other toxic compounds and transport them out of the cell. Metallothioneins are also involved in binding heavy metals, and glutathione S-transferases detoxify a wide range of xenobiotics. Transporters, such as ATP-binding cassette transporters, multidrug and toxin efflux transporters, and oligopeptide transporters, are also crucial in moving xenobiotics out of the cell. An inclusive transcriptome analysis can provide valuable insights into the molecular mechanisms underlying hyperaccumulation and phytoremediation. Transcriptome analysis involves the sequencing and analysis of all the messenger RNA molecules produced by a cell or tissue, which can provide information on gene expression and regulatory mechanisms. This information can help researchers understand how plants respond to xenobiotic stress and identify potential targets for genetic engineering to improve phytoremediation performance [89].

Overall, the use of these aids and techniques can help to enhance the effectiveness of phytoremediation and make it a more viable option for the clean-up of contaminated sites. Like any other remediation technique, phytoremediation must be evaluated case-by-case to determine its effectiveness and safety for a particular area. Factors such as soil characteristics, contaminant type and concentration, climate, and plant species should all be considered before implementing a phytoremediation strategy [53].

## 6. Factors Affecting Phytoremediation Potential

**Figure 6:**
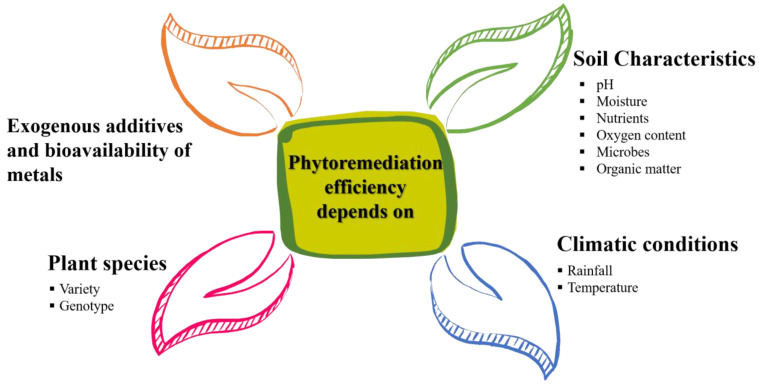
Factors affecting phytoremediation potential.

The accessible part of metals is the fraction plants can take up and is influenced by soil pH, redox potential, and the presence of chelating agents or competing ions, as shown in Figure 6. The inaccessible part of metals is firmly bound to soil minerals and cannot be extracted by plant roots. Hence, it is essential to select appropriate plant species and optimize soil conditions to enhance the bioavailability of metals for effective phytoremediation [44,54]. In addition, chelating agents, such as EDTA or citric acid, can enhance the uptake of metals by plant roots, but they may also increase the risk of metal leaching and contamination of groundwater [44]. The use of chelating agents to alter the bioavailability of strong metals may be harmful to plants and influence their behavior. Along these lines, it is essential to consider the advantages and disadvantages of chelating agents to guarantee that they are safe for the environment and climate. Table 2 shows a compilation of work which has been carried out previously by many researchers on factors which affect the performance of phytoremediation. 

Several factors can affect the potential of phytoremediation to remove contaminants from soil, including:

- Plant species: Different plant species have varying abilities to accumulate and remove contaminants from soil. Hyperaccumulator plants are particularly effective in absorbing heavy metals from soil.
- Contaminant type and concentration: The type and concentration of the contaminant in the soil can affect the plant’s ability to absorb and remove it. Some contaminants, such as heavy metals, can be more difficult to remove than others.
- Soil properties: Soil properties, such as pH, organic matter content, and nutrient availability, can affect the ability of plants to grow and absorb contaminants.
- Climate and weather conditions: Climate and weather conditions, such as temperature, precipitation, and sunlight, can affect plant growth and the rate of contaminant removal.
- Soil moisture: The moisture content of the soil can affect the growth and health of the plants, as well as the availability of the contaminants for uptake.
- Plant growth stage: The growth stage of the plant can affect its ability to absorb contaminants, as well as the biomass produced for removal.
- Duration of treatment: The duration of phytoremediation treatment can affect the effectiveness of contaminant removal. More extended treatment periods may be necessary for some contaminants and soil types.
- Management practices: Proper management practices, such as soil amendments and fertilization, can improve plant growth and the effectiveness of phytoremediation. In addition, the local microbial area in the rhizosphere can improve phytoremediation by influencing the accessibility and versatility of heavy metals in soils [99]. Hence, studying microbial ecology and its interactions with plants and soils is necessary to use phytoremediation effectively. Therefore, it is essential to carefully assess phytoremediation’s potential risks and benefits before its implementation in contaminated sites [100].

## 7. Phytoremediation: Challenges and Difficulties

### 7.1. Application of Phytoremediation Techniques Needs to Be Accelerated

Phytoremediation can be influenced by several factors, as mentioned in the previous section. The biomass production of plants and the accumulation rate of toxins in the plants can affect the time required for phytoremediation. The amount of biomass produced by plants determines the amount of pollutant that can be removed from or stabilized in the soil, and the rate of accumulation of toxins in plants affects the speed at which they can be removed from the site. The slow growth of many hyperaccumulating plants can limit their effectiveness in phytoremediation [101]. Moreover, the remediation of a contaminated site using phytoremediation alone can be a slow process, taking years to even centuries for some metals. The availability and adaptability of foreign substances in the soil, as well as the ability of the plants to retain them, frequently limit the uptake and accumulation of heavy metals in plants. Additionally, the extent and depth of root growth of the plants used for remediation can also limit their performance, as the roots may not cover the entire depth and extent of the contaminated area. Indeed, the time required for phytoremediation to effectively reduce or remove heavy metal contamination can be a significant challenge [54]. Additionally, the root growth of these plants may not reach the full depth of the contaminated area, limiting their effectiveness. As a result, phytoremediation may need to be combined with other remediation techniques, and the overall cost and time involved in the process must be carefully considered [102].

### 7.2. Lack of Effective Methods to Remove Contaminated Biomass

**Figure 7:**
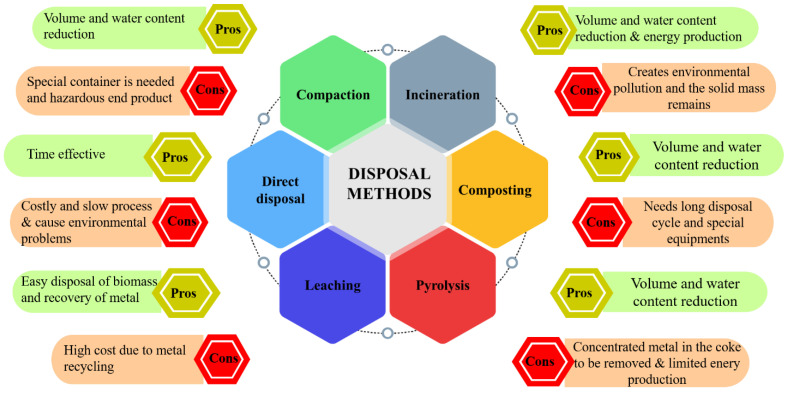
Pros and cons of various disposal methods of polluted biomass after phytoremediation.

Phytoremediation is an environmentally friendly and cost-effective approach that can be used alone or with other remediation techniques. However, phytoextraction can result in contaminated biomass, which requires proper disposal to prevent further environmental pollution. It is essential to consider the potential environmental impacts of both the phytoremediation process itself and the removal of the resulting contaminated biomass [103]. The six standard methods for disposing of contaminated biomass (composting, landfilling, pyrolysis, leaching, cremation, and direct disposal) each have advantages and disadvantages. They must be carefully evaluated based on the specific circumstances of each site, as shown in Figure 7. Soil treatment involves using microorganisms to break down the contaminants in biomass, while compaction compresses biomass into a dense, stable material. Pyrolysis is a process of thermal degradation that converts biomass into solid, liquid, and gas fractions [104]. Leaching relies on the ability of soluble heavy metals to migrate through a medium, and cremation involves thermal degradation of biomass into treatable-metal-containing ash. Direct disposal is not recommended due to its potential environmental risks. Further research is needed to develop effective and environmentally friendly methods for disposing of contaminated biomass generated from phytoremediation [105]. 

## 8. Advancements in Research to Address the Problems and Challenges

### 8.1. Techniques Used to Increase the Effectiveness of Phytoremediation

Genetic engineering can enhance plants’ phytoremediation potential by introducing or modifying genes that can improve their ability to absorb and detoxify pollutants. This involves introducing genes that can increase plant metal uptake, transport, and detoxification [55]. However, this approach raises ethical concerns and regulatory issues about releasing genetically modified organisms (GMOs) into the environment. Another method is to use a combination of different plant species, known as phytococktails, to remediate multiple contaminants simultaneously [14]. This approach can increase the overall effectiveness of phytoremediation, as other plant species have additional metal uptake and accumulation capabilities. Lastly, improving the phytoremediation process can also be achieved by optimizing growth conditions, for instance, by adjusting soil pH, nutrient levels, and water availability [101]. This can enhance plant growth and metal uptake, thereby increasing phytoremediation efficiency. However, this approach may require additional resources and maintenance efforts. Overall, using different strategies and techniques can significantly improve the effectiveness of phytoremediation for contaminated soils and water bodies. However, each approach has its advantages and limitations and should be carefully evaluated based on the specific context and goals of the remediation project [45].

Agronomic measures, such as compost application, water guidelines, and further developed culturing procedures, can further enhance the proficiency of the phytoremediation of heavy metal contamination [106]. In addition, phytoremediation time can be decreased by various means, such as genetic engineering, soil amendments, and plant growth regulators. A plant’s capacity to accumulate and withstand heavy metals can be enhanced genetically, and soil enhancements, such as biochar and fertilizers, can enhance soil quality and increase the availability of nutrients for plants. Plant growth regulators can promote plant growth and boost biomass production, leading to higher metal uptake by plants [107]. However, it is essential to note that using some of these methods, such as chelating agents and genetic engineering, may negatively impact the environment and human health if not used properly. It is, therefore, necessary to carefully evaluate and monitor these methods’ effectiveness and potential risks before their widespread application in phytoremediation [48].

This information will help identify transporters and ion channels involved in metal uptake and homeostasis, which can be targeted for genetic engineering to enhance phytoremediation efficiency. Genetic engineering can also introduce metal-resistant genes from other organisms into plants or modify plant enzymes involved in metal uptake and detoxification processes [54]. However, using genetically modified plants in phytoremediation still faces regulatory and ethical challenges, and more research is needed to ensure their safety and effectiveness. Hereditary designing depends on the *in vitro* presentation of unfamiliar qualities into beneficiary cells to change their unique genetic attributes and acquire new assortments of existing items or produce new ones. The responses of different heavy-metal-pressure in different plants are determined through sub-atomic and genetic tests using the attributes of absorption abilities and resilience of hyperaccumulators [54].

**Figure 8:**
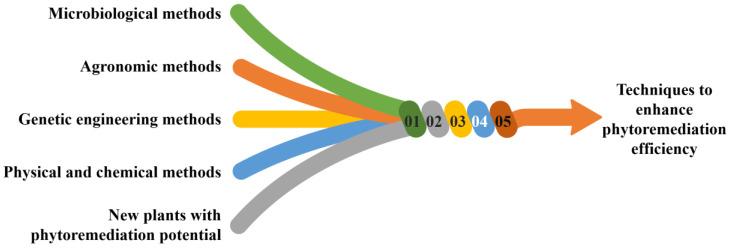
Various techniques to enhance phytoremediation efficiency.

Selecting suitable plant species depends on several factors, such as the type and concentration of contaminants, soil, climatic conditions, and phytoremediation goals. For example, some plants are better suited to the remediation of organic contaminants, while others are more effective in removing heavy metals. In addition to exploring new plant species, developing genetically modified plants can expand the range of phytoremediation options. Genetic engineering can enhance plants’ metal-accumulating capacity, increase their tolerance to toxic contaminants, and change their root systems so that they capture and store contaminants better [45,51]. Overall, continued research and development of phytoremediation technology will be necessary to increase its effectiveness, expand the range of plant species and genotypes available, and address the unique challenges of different contaminants and environmental conditions [108]. A schematic representation of various techniques to enhance phytoremediation efficiency is shown in Figure 8. 

### 8.2. Disposal of Harmful Plant Waste after Phytoremediation

Pyrolysis is an effective method for changing the form of heavy metals in biomass and making them more stable. The pyrolysis temperature is also critical, as higher temperatures result in more substantial stability. Pyrolysis can also recover heavy metals from contaminated biomass, such as *Pteris vittata* [54]. Heavy metals exist in various forms and quantities in plants, such as lead (Pb), predominantly in the form of soluble inorganic and amino acid salts in striped seaweed. In contrast, cadmium (Cd) mainly exists as gelatin and protein in tea plants, *Panicularia paniculata*, and impatiens resin [109]. It also exists as heavy metal phosphate and oxalate, which are insoluble in water, grain roots, gelatin, and protein binding in grain malt. Therefore, it is essential to investigate the different forms of heavy metals in plants to determine the appropriate removal techniques [110]. Disposal of plant waste after phytoremediation is a critical step in the process. The plant biomass accumulating heavy metals must be carefully handled and disposed of to avoid causing further contamination.

The harvested plant material can sometimes be incinerated to recover any accumulated metals in the biomass. However, incineration is not always practical or cost-effective and may release pollutants into the air. Alternatively, the plant material can be landfilled or deposited in isolated hazardous waste sites. However, the disposal of biomass in landfill or hazardous waste sites can lead to long-term environmental pollution. To mitigate this, the harvested plant material can be used for other purposes, such as composting or bioenergy production. In composting, the plant biomass can be mixed and ‘diluted’ with other organic materials and allowed to decompose to produce a nutrient-rich soil amendment. The resulting compost can be used for agriculture, horticulture, or landscaping. In bioenergy production, plant biomass can yield energy through various methods, such as anaerobic digestion, pyrolysis, or combustion. This approach can help reduce the reliance on fossil fuels and provide a renewable energy source [32].

## 9. Challenges and Future Recommendations

**Figure 9:**
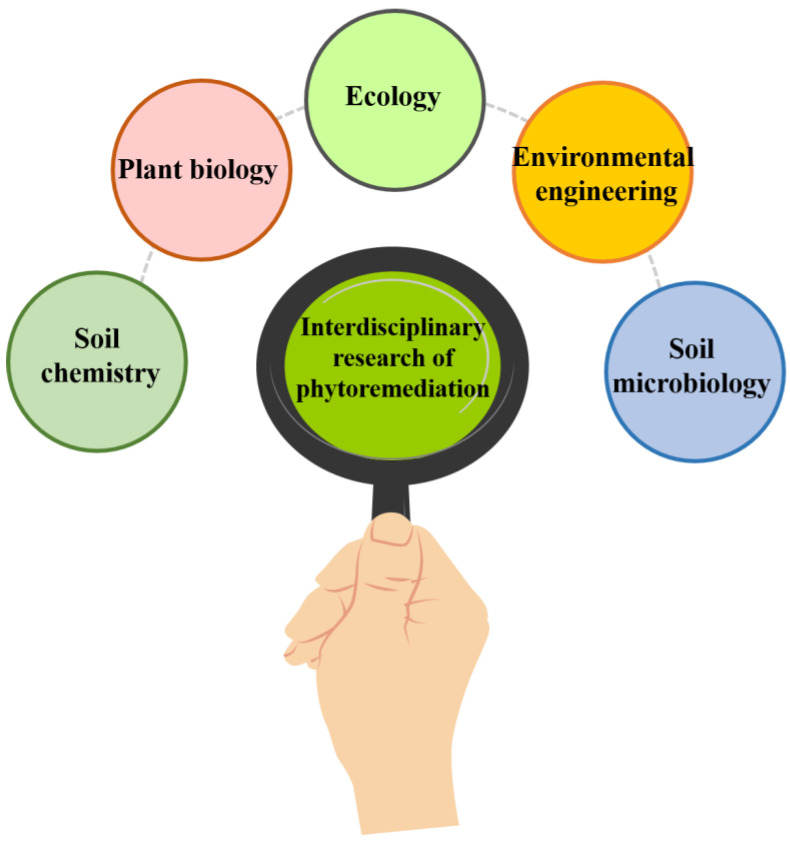
Interdisciplinary research on phytoremediation.

Even though phytoremediation is a successful effective strategy for removing pollutants from the environment, it has a few limitations and disadvantages on which limited knowledge exists since most of the exploration has been carried out in controlled environments. If the level of toxins in the soil is too high, this can affect the plant’s growth and ability to remediate the site. Additionally, some plant species may be more suitable for certain types of contamination than others and selecting the wrong plant species could lead to lower phytoremediation efficiency. Furthermore, there is a risk of plant uptake of contaminants, which could lead to potential health risks if animals or humans consume the contaminated plant material. Finally, the economic feasibility of phytoremediation can be a limiting factor, as the costs associated with plant growth, monitoring, and maintenance can be high [54,111]. Interdisciplinary research on phytoremediation to enhance efficiency is shown in Figure 9. Disadvantages include the biomass after remediation being toxic and, if it is burned, the ash also being toxic, as well as the toxicity of the end products of remediation plant composting.

Phytoremediation is a multidisciplinary field involving various scientific disciplines that aims to develop and improve phytoremediation technology. Interdisciplinary research is essential for enhancing phytoremediation efficiency and addressing the complex environmental problems associated with soil contamination. Some scientific disciplines involved in phytoremediation research include botany, plant physiology, soil science, microbiology, chemistry, aquatic science, and environmental engineering. Combining the knowledge and expertise of scientists from these disciplines makes it possible to develop more effective phytoremediation strategies. For example, soil scientists can provide valuable information on the physical and chemical properties of the soil, which can affect plant growth and the uptake of contaminants. Environmental engineers can provide insight into the design and implementation of phytoremediation systems, including selecting appropriate plants and optimizing environmental conditions. Botanists and plant physiologists can provide information on the uptake, translocation, and detoxification of contaminants by plants, and microbiologists can give insight into the role of soil microorganisms in facilitating phytoremediation processes. Aquatic biologists would help finding suitable plants for removal of specific contaminants and their efficacy in hydric and under water soils. Furthermore, interdisciplinary research can help to identify the limitations and challenges associated with phytoremediation and develop solutions to overcome these challenges. For instance, molecular biology techniques can be used to develop genetically engineered plants to increase their ability to accumulate and detoxify contaminants, while nanotechnology can enhance the uptake and transport of contaminants by plants [6].

Phytoremediation is a promising method for the remediation of heavy metals from polluted soil, but it still has some limitations and challenges that must be addressed:

- It is a slow process which may take several years to achieve significant results, especially in highly contaminated soils. The time required for remediation depends on several factors, such as the type and concentration of contaminants, the plant species used, soil properties, and environmental conditions.
- It can sometimes be less effective due to some hyperaccumulator plants’ slow growth rates and lower levels of biomass production. These factors can limit the amounts of heavy metals removed from contaminated soil within a given period. Additionally, some plants may only accumulate specific types of heavy metals, which may not be the most predominant contaminants in the soil. There may also be contaminants with lower activation abilities or plants with lower absorption potential because of a few firmly bound metal particles. Thus, there is a risk of only partial removal of pollutants from the contaminated site in the absence of appropriate consideration.

Therefore, it is crucial to conduct field studies to validate the effectiveness of phytoremediation techniques in real-world conditions. Field studies can help to identify the most effective plant species, planting densities, soil amendments, and other factors that can maximize the removal of heavy metals from contaminated soils. Moreover, field studies can also provide valuable information about the long-term sustainability and cost-effectiveness of phytoremediation as a remediation technology [11,45,112]. Integrating scientific knowledge from different fields is essential to advance phytoremediation technology further. Additionally, more research is needed to assess the potential environmental impacts of the byproducts and toxins produced during the phytoremediation process and ways to enhance the growth of hyperaccumulating species in highly contaminated areas. The exploration of commercial opportunities through market niches of green roof construction, phytomining, and plant-based bioengineering projects also presents a potential avenue for further development and application of phytoremediation technology [112]. By incorporating different advancements from various disciplines, inter-disciplinary cooperation should be strengthened to speed up restoration, lower reclamation costs, and improve restoration outcomes. The cleaning effectiveness of heavy-metal-contaminated substrates should be improved through the optimization of rebuilding techniques, reduction in maintenance process duration, cost reduction, and avoidance of auxiliary impurities in the plant recovery cycle.

## 10. Conclusions

Phytoremediation has excellent potential as a plant-mediated remediation technology. However, its development and application have been limited by low remediation efficiency and ineffective disposal methods available for contaminated biomass. While some research has been conducted to improve phytoremediation efficiency, more investment is needed to make it a practical and effective solution for large-scale remediation projects. This will enable the technology to move from the lab to practical applications on a large scale. While there are constraints, for example, long improvement times and reliance on the specific environment and plant development, a considerable number of these restrictions can be overcome by legitimate planning and appropriate species determination. However, more research is needed to demonstrate the effectiveness of phytoremediation technology and increase its application. Combining different remediation processes, such as phytoremediation and soil amendment, can also support the effective treatment of heavy metal pollution. Additionally, genomic, proteomic, and metabolic studies can provide valuable information on the mechanisms of plants’ heavy metal uptake, transport, and detoxification. This information can be used to develop more effective phytoremediation strategies and overcome existing limitations.

Furthermore, use of the existing microbial population can help expand the absorptive capacity of roots by corroding common impurities and improving metal uptake. Genetically engineered plants have the potential to enhance phytoremediation by increasing plant growth rates and over-expressing genes that control metal uptake and transport. Phytoremediation is also more cost-effective than other remediation methods, with studies showing 5–13 times lower costs. Additionally, researchers can explore the secondary benefits of phytoremediation, such as linking it to biomass production that can be used for bioenergy, feedstock for pyrolysis and biofortified products, and carbon sequestration, which can contribute to phytomanagement. Studying phytoremediation at molecular-genetic and nano levels is also possible and promising.

## Figures and Tables

**Table 1:** Potential plant species for heavy metal remediation.

S. No.	Plant Species	Contaminants Removed	Ref.
1	*Brassica juncea* L.	Cd, Cu, Zn	[57,58,59]
2	*Populus* sp.	Cd	[60]
3	*Helianthus annuus*	Zn	[61]
4	*Melica jacquemontii Poaceae*	Fe	[62]
5	*Medicago sativa, Brassica nigra*	Pb	[63]
6	*Eleocharis acicularis*	Cu	[64]
7	*Lemna minor*	Pb, Cd, Ni, Cr	[65]
8	*Brassica rapa* L.	U	[66]
9	*Alyssum murale, Berkheya coddii*	Ni	[67]
10	*Azolla filiculoides*	Hg (II), Pb (II)	[68]
11	*Jatropha curcas*	Al, Cd, Fe, Cr, Pb, Zn, Ni, Cu	[69]
12	*Viola bashanensis*	Zn	[70]
13	*Aeollanthus subacaulis*	Cu	[71]
14	*Oryza sativa*	Cd, Zn, Fe, Cu, Pb, Cr, Mn	[72,73]
15	*Schima superba*	Mn	[74]

**Table 2:** Factors affecting the performance of phytoremediation in earlier studies.

Plants	Target Medium	Inducing Factor	Observation	Ref.
*Helianthus tuberosus*	Soil	Based on metal and its concentration and pH	*Helianthus tuberosus* showed adequate growth in the presence of minor metal fixations and a pH range of 5 to 6. With the addition of metals in the soil at pH 5, the grouping of metals in shoots grew.	[90]
*Lemna valdiviana*	Water	pH (3.94–9.02) P (0–0.14 mmol·L^−1^) N (0.09–13.71 mmol·L^−1^)	*Lemna valdiviana* collect more significant amounts of As (1190 mg kg^−1^) when the pH is between 6.30 and 9, the P concentration is 0.05 mmol L^−1^, and the N concentration is 7.90 mmol L^−1^.	[77]
*Ulva ohnoi*	Water	Salinity and temperature	*Ulva ohnoi* continued to develop favorably between 18 and 25 °C, S35. The focus factor with the highest value was 81.30% of Cd added at 0.63 gL^−1^ to 18 C and S15.	[91]
*Vicia faba* L.	Soil	Genotype	According to all indications, the genotype LXYC was the most suitable one for phytoremediation in soil that had been moderately or slightly depleted in Pb and Cd.	[92]
*Arabidopsis thaliana* and *Populus alba*	Soil/water	Genetic modification	Plants that communicate with ScZRC1, such as poplar and *A. thaliana*, could accumulate more Zn.	[93]
*Festuca arundinacea*	Soil	Planting density	At D20, the biomass and Cd accumulation peaked (13.30 g Cd m^−2^).	[94]
*Solanum nigrum* L.	Soil	Carbon nanotubes with multiple walls	At 5.23% to 27.97%, multi-walled carbon nanotubes could increase plant biomass.	[95]
*Bidens pilosa* L.	Soil	Activated carbon (biochar)	Under treatments containing 100 and 200 mg kg^−1^ biochar, respectively, the accumulation of Cd increased by 16.44 and 39.37%.	[96]
*Pennisetum hybridum*	Soil	Digestate	The growth in digestate could increase compact Cd fixation in the stems (7.94–42.39%), leaves (12.53–74.11%), and roots (18.59–57.94%).	[97]
*Willow*	Soil	Urea	Under the treatment with Cd and urea, the individual Cd convergences of the roots, xylem, bark, and leaves were 8.30, 8.15, 26.79, and 33.04 mg kg^−1^.	[98]

## Data Availability

Data will be given upon request.
